# Developing and validating a prognostic nomogram for ovarian clear cell carcinoma patients: A retrospective comparison of lymph node staging schemes with competing risk analysis

**DOI:** 10.3389/fonc.2022.940601

**Published:** 2022-11-09

**Authors:** Yiling Li, Lin Xiu, Mingyuan Ma, Samuel Seery, Xiaoying Lou, Kexin Li, Yue Wu, Shuang Liang, Yuxin Wu, Wei Cui

**Affiliations:** ^1^ State Key Laboratory of Molecular Oncology, Department of Clinical Laboratory, National Cancer Center/National Clinical Research Center for Cancer/Cancer Hospital, Chinese Academy of Medical Sciences and Peking Union Medical College, Beijing, China; ^2^ Department of Gynecology Oncology, National Cancer Center/National Clinical Research Center for Cancer/Cancer Hospital, Chinese Academy of Medical Sciences and Peking Union Medical College, Beijing, China; ^3^ Department of Statistics, Department of Electrical Engineering and Computer Sciences, University of California, Berkeley, United States; ^4^ Faculty of Medicine and Health, Division of Health Research, Lancaster University, Lancaster, United Kingdom

**Keywords:** ovarian clear cell carcinoma, lymph node staging, competing risk model, prognosis, nomogram

## Abstract

**Purpose:**

Lymph node (LN) involvement is a key factor in ovarian clear cell carcinoma (OCCC) although, there several indicators can be used to define prognosis. This study examines the prognostic performances of each indicator for OCCC patients by comparing the number of lymph nodes examined (TNLE), the number of positive lymph nodes (PLN), lymph node ratio (LNR), and log odds of metastatic lymph nodes (LODDS).

**Methods:**

1,300 OCCC patients who underwent lymphadenectomy between 2004 and 2015 were extracted from the Surveillance Epidemiology and End Results (SEER) database. Primary outcomes were Overall Survival (OS) and the cumulative incidence of Cancer-Specific Survival (CSS). Kaplan–Meier’s and Fine-Gray’s tests were implemented to assess OS and CSS rates. After conducting multivariate analysis, nomograms using OS and CSS were constructed based upon an improved LN system. Each nomograms’ performance was assessed using Receiver Operating Characteristics (ROC) curves, calibration curves, and the C-index which were compared to traditional cancer staging systems.

**Results:**

Multivariate Cox’s regression analysis was used to assess prognostic factors for OS, including age, T stage, M stage, SEER stage, and LODDS. To account for the CSS endpoint, a proportional subdistribution hazard model was implemented which suggested that the T stage, M stage, SEER stage, and LNR are all significant. This enabled us to develop a LODDS-based nomogram for OS and a LNR-based nomogram for CSS. C-indexes for both the OS and CSS nomograms were higher than the traditional American Joint Committee on Cancer (AJCC), 8th edition, staging system. Area Under the Curve (AUC) values for predicting 3- and 5-year OS and CSS between nomograms also highlighted an improvement upon the AJCC staging system. Calibration curves also performed with consistency, which was verified using a validation cohort.

**Conclusions:**

LODDS and LNR may be better predictors than N stage, TNLE, and PLNs. For OCCC patients, both the LODDS-based and LNR-based nomograms performed better than the AJCC staging system at predicting OS and CSS. However, further large sample, real-world studies are necessary to validate the assertion.

## Introduction

Epithelial ovarian carcinoma (EOC) is one of the most common gynecological tumors, which has high mortality once metastasizing. Ovarian cancer accounts for 2.5% of all female malignant tumors, but the death rate represents 5% of all tumor-related mortality which ranks fifth among female cancers (1). Ovarian clear cell carcinoma (OCCC) is a specific pathological EOC type that has a particularly high prevalence across Asia, and especially in Japan (2). As a relatively rare subtype of ovarian cancer, this only accounts for 5% of all EOCs. Clear cell carcinoma is characterized by the young age of cases and platinum resistance. However, advanced clear cell carcinoma also has dismal prognosis and a high frequency of associated deaths, largely due to its resistance to chemotherapeutics (3–5). At present, there is insufficient evidence to develop accurate prognostics strategies for OCCC and therefore, it is of paramount importance that we explore pathological characteristics and prognostic factors to predict outcomes. This will enable us to develop interventions which combat this aggressive malignancy and improve survival.

Lymph node status is a key indicator used for assessing metastases, in terms of recurrence, survival, and for scheduling adjuvant therapies for OCCC patients. Lymph node status is also a key factor used to guide postoperative therapeutics planning (6). Likewise, the N stage system, which classifies tumors according to the American Joint Committee on Cancer (AJCC), and the Union for International Cancer Control (UICC) Tumor Node Metastasis (TNM) classification, are widely used to determine the presence (or absence) of lymph node metastases (7, 8). While the N stage system is reproducible, it may reduce the likelihood of stage migration which can misguide treatment practice. Some staging systems have been established, including the Total Number of Lymph Nodes Examined (TNLE), the number of Positive Lymph Nodes (PLN), Lymph Node Ratio (LNR), and Log ODDS of positive nodes (LODDS), in order to accurately describe lymph node status (9, 10). However, researchers have attempted to develop alternative lymph node staging systems which more accurately predict survival for patients with a number of different cancers. For example, Ye et al. found that LODDS staging, for patients with esophageal carcinoma who have received neoadjuvant therapies, performed better than both the positive Lymph Node Ratio (LNR) and the N descriptor (11). Han et al. also reported that LODDS and LNR enhanced the discriminability and goodness of fit in predicting survival for stage IV rectal cancer patients, compared to the N stage system (12). However, few studies have compared all nodal staging systems including TNLE, PLNs, LNR, and LODDS, and none have comprehensively compared these for ovarian clear cell carcinoma patients.

Of course, competing risks occur commonly in cancer research, and OCCC is no exception. For example, the use of Bevacizumab, a targeted drug prescribed for ovarian cancer, has been associated with serious and sometimes fatal complications, including gastrointestinal perforations (13), cardiac toxicity, vascular thromboembolism (14), etc. These competing events might influence the probability of observed patient deaths. Additionally, Kaplan-Meier’s analysis and Cox’s regression modeling can generate biased results if researchers do not account for competing events (15, 16). Therefore, a competing risk model should be taken into consideration when analyzing cancer prognoses. Adopting this approach could also enable us to develop a nomogram which could improve outcomes for patients from diverse ethnic backgrounds and with heterogeneous clinical characteristics. To the best of our knowledge, although few studies that examined the prognostic potential of lymph node status in ovarian cancer, there is no competing risk nomogram research which uses subdistribution-based, proportional hazard modeling for OCCC subtype patient prognosis (17). Therefore, we aimed to compare different nodal staging systems to identify the most effective and to find out which lymph node staging system is the best prognostic indicator for OS and CSS. Ultimately, we hope to develop and validate a nomogram which incorporates prognostic factors, to improve OCCC outcomes.

## Patients and methods

### Data collection

Anonymized data were extracted from the Surveillance Epidemiology and End Results (SEER) database which includes data from 18 population-based registries from the 1^st^ January 2004 to the 31^st^ December 2015. Inclusion criteria were used to identify eligible individuals, including: OCCC as the first and only primary diagnosis, and OCCC confirmed through pathological examination.

Histopathological types were defined according to the International Classification of Diseases for Oncology, 3rd edition (ICD-O-3) morphological codes, which include Clear Cell Adenocarcinoma (8310/3), Clear Cell Adenocarcinofibroma (8313/3), Clear Cell Cystadenocarcinoma (8443/3), and Clear Cell Cystic tumors, and other malignancies (8444/3) (18).

Patients were excluded for reasons, including unknown follow-up and survival information; having two (or more) primary tumor lesions; and having unknown lymph node status or when there were other unknown clinicopathological data.

This retrospective study also extracted demographics such as age, ethnic origin, year of diagnosis, and clinical data including clinical TNM stage, SEER stage of neoplasia, pathological data i.e., tumor size, histological pathology, grade, and lymph nodal status, and survival outcomes regarding follow-up e.g., survival period, OS, and CSS rates.

### Optimal cutoff values for lymph node staging schemes

Patients were assigned according to the current SEER staging system, and lymph node status was assessed using histological parameter pN (TNM 8th edition). In addition to the continuous variable i.e., PLN, we used both continuous and categorical variables to analyze TNLE, LNR, and LODDS, with cutoff values calculated using X-tile software, version 3.6.1 (19).

TNLE was classified into three groups, TNLE 1:1-3; TNLE 2: 4-8; TNLE 3: >8. LNR was defined as the number of positive lymph nodes divided by the number of total examined lymph nodes which were stratified into two groups: LNR1 ≤ 0.04 and LNR2>0.04. LODDS was defined as log (the number of PLNs +0.5)/(the number of negative nodes +0.5) which were then categorized into either LODDS0≤-1.160, -1.160< LODDS1≤-0.640, LODDS2>-0.640.

### Development and validation of the nomogram model for OS and CSS

Initial survival analysis was performed using Kaplan-Meier curves, and the differences between curves were verified using the log-rank test for OS. The predictive efficacy of four lymph node staging schemes were compared using the Akaike information criterion (AIC) and the C-index.

The LODDS staging system with maximal accuracy was further identified using Cox’s multivariate regression analysis with other significant independent prognostic indicators for OS. We treated death from cancer-specific reasons and death from other reasons as two competing events.

Fine and Gray’s test and Cumulative Incidence Function (CIF) was implemented to identify significant univariate results. Then, proportional subdistribution hazard modeling was used to estimate the impact of significant CSS variables.

Nomograms based on the lymph node system for the OCCC case were constructed to predict 3- and 5-year prognosis, both in overall and cancer-specific survival rates. Nomogram performance was verified internally through the training cohort and then, externally using a validation cohort based on the C-index, AUC, and calibration curves.

### Statistical analysis

All statistical analyses were carried out using SPSS (version 24.0) and R (version 3.6.0). P values <0.05 were considered statistically significant.

## Results

### Characteristics and prognosis of patients with ovarian clear cell carcinoma

**Figure 1:**
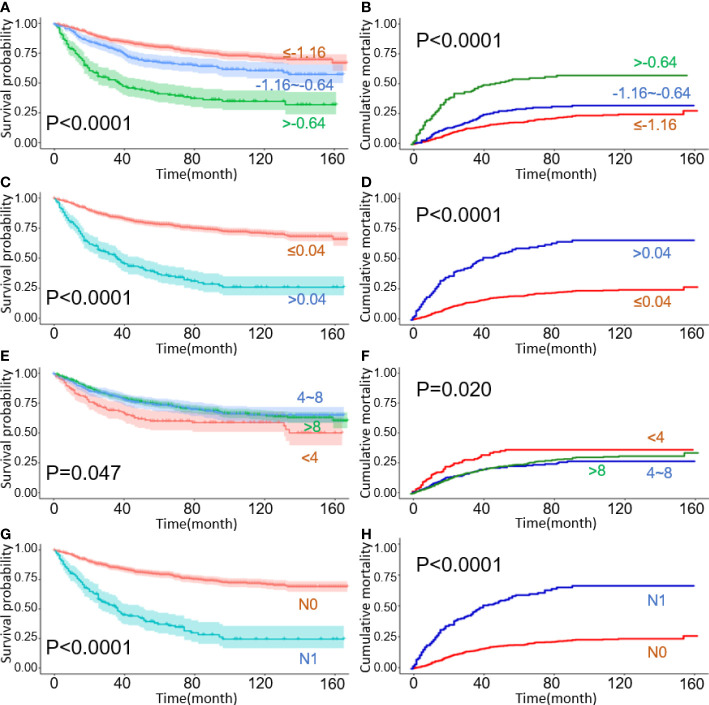
Kaplan–Meier curves for overall survival with cumulative incidence curves for cancer-specific deaths, stratified by lymph node staging schemes: **(A, B)** LODDS; **(C, D)** LNR; **(E, F)** TNLE; **(G, H)** N stage.

A total of 1,300 ovarian clear cell carcinoma patients who underwent lymphadnectomy, including pelvic lymph node resection and/or para-aortic lymph node resection between 2004 and 2015 were enrolled with median age of 55.3 years old at diagnosis. Demographics and clinicopathological features for all patients have been provided in **Table 1** (below). The selection process has also been summarized and provided in the supplementary materials as **Figure 1**.

**Table 1:** Clinical characteristics in prognostic results and univariate cox analysis for OS.

Variables	N = 1300	Overall survival		OS Univariate analysis	
		3-year OS (%)	5-year OS (%)	HR (95% CI)	P value	
**Age at diagnosis, years**					< 0.001*	
<57	717 (55.2%)	80.8	72.4	Reference		
57-67	423 (32.5%)	69.0	53	1.220 (0.975-1.529)	0.083	
>67	160 (12.3%)	88.6	85.2	2.051 (1.565-2.690)	< 0.001*	
**Year of diagnosis**					0.334	
2004-2010	513 (39.5%)	93.0	88.1	Reference		
2011-2015	787 (60.5%)	80.3	72.7	0.902 (0.733-1.112)	0.335	
**Race**					0.034*	
White	973 (74.8%)	78.6	72.4	Reference		
Black	52 (4.0%)	69.0	53.0	1.675 (1.086-2.585)	0.02*	
Other	275 (21.2%)	81.7	73.9	0.900 (0.698-1.162)	0.42	
**Grade**					0.071	
I	19 (1.46%)	83.5	75.2	Reference		
II	151 (11.6%)	84.3	76.0	0.769 (0.325-1.823)	0.552	
III	735 (56.5%)	76.4	69.2	1.144 (0.509-2.572)	0.745	
IV	395 (30.4%)	81.1	74.7	0.925 (0.406-2.108)	0.853	
**Tumor size, cm**					0.035*	
<8.5	355 (27.3%)	81.6	76.5	Reference		
≥8.5	945 (72.7%)	78.0	70.0	1.287 (1.018-1.628)	0.035*	
**T stage**					< 0.001*	
1	877 (67.5%)	90.8	85.1	Reference		
2	189 (14.5%)	71.3	65.8	2.476 (1.857-3.301)	< 0.001*	
3	234 (18.0%)	41.4	27.7	7.862 (6.287-9.832)	< 0.001*	
**M stage**					< 0.001*	
0	1236 (95.1%)	81.2	74.7	Reference		
1	64 (4.9%)	36.2	18.7	5.893 (4.373-7.942)	< 0.001*	
**SEER stage**					< 0.001*	
Localized	471 (36.2%)	93.4	89.2	Reference		
Regional	573 (44.1%)	83.0	77.3	2.047 (1.518-2.761)	< 0.001*	
Distant	256 (19.7%)	43.2	28.5	10.066 (7.525-13.464)	< 0.001*	
**LODDS classification**					< 0.001*	
≤-1.160	841 (64.7%)	85.3	79.8	Reference		
<-1.160, ≥-0.640	292 (22.5%)	76.8	66.7	1.616 (1.265-2.064)	< 0.001*	
>-0.640	167 (12.8%)	50.7	41.0	*3.811 (2.992-4.885)*	< 0.001*	
**LNR classification**					< 0.001*	
≤0.04	1111 (85.5%)	84.1	78.0	Reference		
>0.04	189 (14.5%)	49.1	35.8	3.999 (3.222-4.963)	< 0.001*	
**TNLE classification**					0.005*	
≤4	156 (12.0%)	68.2	59.9	Reference		
< 4, ≥8	295 (22.7%)	80.2	73.2	0.640 (0.461-0.888)	0.008*	
>8	849 (65.3%)	80.3	73.8	0.637 (0.482-0.843)	0.002*	
***PLNs (median, range)***	0 (0,97)	84.3	78.4	1.021 (1.013-1.029)	< 0.001*	
**LODDS *(median, range)***	-1.36 (-2.29,2.29)	84.6	80.9	2.026 (1.798-2.283)	< 0.001*	
**LNR *(median, range)***	0 (0,1)	84.3	78.4	8.649 (6.196-12.072)	< 0.001*	
**TNLE *(median, range)***	13 (1,97)	71.6	61.4	1.000 (0.993-1.006)	0.934	

Other, American Indian/AK Native, Asian/Pacific Islander; SEER, surveillance, epidemiology, and end results; LODDS: log ((the number of PLNs +0.5)/(the number of negative nodes +0.5)); LNR: the lymph node ratio; TNLE: total number of lymph nodes examined; PLNs: the number of positive lymph nodes; OS, overall survival; P value: log-rank test. * means statistically significant.

In terms of survival, the mean follow-up was 64.9 months (range 0–167 months) and 29.9% of this patient sample (388/1300) died. 25.8% of OCCC patients (355/1300) died of cancer-specific reasons. According to the optimal cut-off value generated using X-tile software, we divided age at diagnosis into three groups: 18-57, 58-67, and >67 years old. The tumor size was divided into two groups, 72.7% were 8.5 cm or more.

In 1.5% of cases, the tumor was well-differentiated (G1), in 11.6% moderately differentiated (G2), in 56.5% poorly differentiated (G3), and in 30.4% undifferentiated (G4). The most frequent SEER stage type was the regional stage (44.1%), followed by the localized (36.2%), and the distant stage (19.7%). With an increased age at diagnosis, tumor size, T stage, M stage, and SEER stage, OCCC patients received significantly worse prognoses *(*all with *p <*0.05).

For LN status, the median number of harvested lymph nodes was 13 (range 1-97), around an average of 16.84. TNLE was classified into three groups as follows: TNLE 1, TNLE 2, and TNLE 3. LNR was classified into two groups, LNR1 (n = 1,111), and LNR2 (n = 189). The value of LODDS was distributed from -2.290 to 2.290 which was classified into three LODDS groups, LODDS1 (n = 841), LODDS2 (n = 292), and LODDS3 (n = 167).

The probability of survival decreased with LNR and LODDS increments, except for the TNLE classification. For example, the 5-year OS reduced from 79.8% for LODDS1 to 41.0% for LODDS3, and the 5-year OS of LNR 1, LNR 2 with 78.0%, and 35.8%, respectively. However, the inverse also appears to occur in the TNLE lymph node system. Mortality risk decreased as the number of elevated LNs increased. The 5-year OS in TNLE 1, TNLE 2, and TNLE 3 were 68.2%, 80.2%, and 80.3%, respectively.

### Survival analysis

Survival analyses were conducted according to demographics and clinical variables using Kaplan-Meier curves, with differences between curves assessed using log-rank tests for OS. In terms of competing risks, CIF curves and Gray’s testing were implemented to the study variables according to 3-, 5- and 8-year CIF values for CSD and DOC.

**Figure 2:**
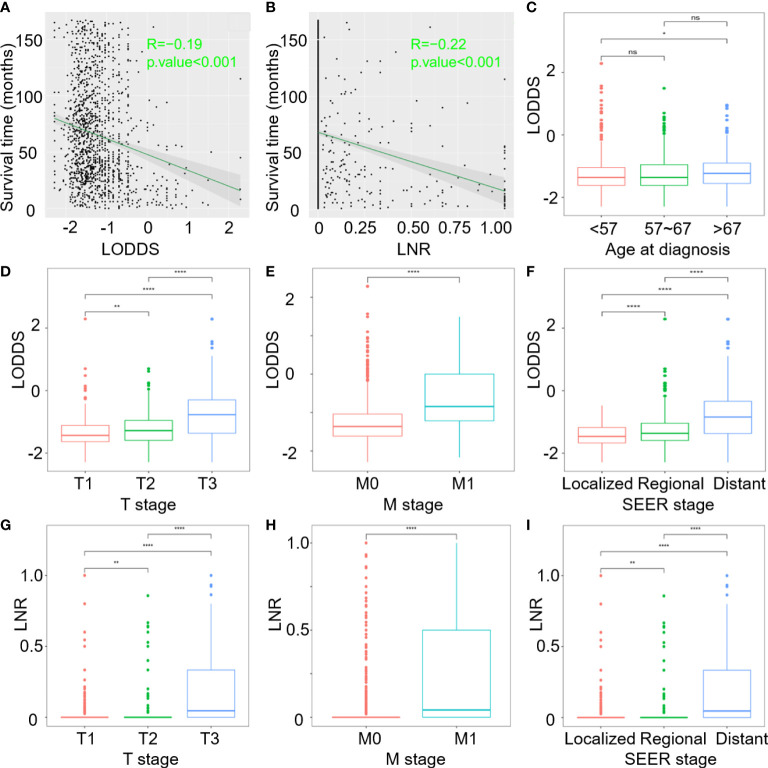
Correlation analysis of: **(A)** Survival time and continuous LODDS; **(B)** Survival time and continuous LNR; **(C–F)** LODDS with age at diagnosis, T stage, M stage, and SEER stage; **(G–I)** LNR with T stage, M stage and SEER stage. (ns, P>0.05; *P < 0.05; **P < 0.01; and ****P < 0.0001).

Significant survival differences were observed across different LN staging systems including traditional N stage, LODDS, LNR, and TNLE shown in **Figure 1**. In patients with the N1 stage, higher LODDs values and LNR significantly correlated with poor prognosis both in OS and CSS groups (all log-*rank p* < 0.05), as listed in **Figure 1A–F**, respectively. Kaplan-Meier survival curves and the CIF curves for demographics and clinicopathological features respectively for OS and CSS analysis have been provided in the supplementary materials as **Figure 2**.

Univariate Cox’s regression analyses predicting OS and Gray’s test predicting CSS were performed to determine which factors are statistically significant (*p* < 0.05). Age at diagnosis, T stage, M stage, SEER stage, classification of TNLE, LNR, and LODDS, and continuous PLNs, LNR, and LODDS values were significant prognostic factors for both OS and CSS (**Tables 1**, **2**), while the year of diagnosis and continuous TNLE variables were influential to neither OS nor CSS (*p* > 0.05).

**Table 2:** The cumulative incidences for CSD and DOC.

Variables	Cause-specific death				Death due to other causes			
	3-year (%)	5-year (%)	Gray’s test	P value	3-year (%)	5-year (%)	Gray’s test	P value
**Age at diagnosis, years**	** **		6.57	0.038*	** **		30.36	< 0.001*
<57	17.86	23.05		** **	1.31	2.06		** **
57-67	18.34	25.98			1.44	2.04		
>67	25.87	31.76			4.54	7.31		
**Year of diagnosis**	** **		1.41	0.234	** **		0.31	0.560
2004-2010	20.99	26.75		** **	1.77	2.56		** **
2011-2015	17.64	23.90			1.73	2.83		
**Race**			4.91	0.086			0.63	0.731
White	19.17	24.62		** **	1.79	2.62		** **
Black	24.55	40.51			6.48	6.48		
Other	17.40	23.84			0.75	2.27		
**Grade**			8.07	0.045*			1.52	0.676
I	10.53	24.84		** **	0.00	0.00		** **
II	13.07	19.63			2.02	2.84		
III	21.44	27.62			1.97	2.83		
IV	17.15	22.35			1.32	2.53		
**Tumor size, cm**			3.57	0.059			0.88	0.349
<8.5	16.79	20.77		** **	1.15	1.87		** **
≥8.5	19.81	26.66			1.97	3.00		
**T stage**			332.13	< 0.001*			4.91	0.086
1	7.99	12.86		** **	0.94	1.74		** **
2	26.13	29.50			2.15	3.52		
3	54.05	66.06			4.41	5.45		
**M stage**			116.45	< 0.001*			1.78	0.183
0	16.82	22.43		** **	1.76	2.64		** **
1	61.61	77.38			1.63	3.67		
**SEER stage**			334.27	< 0.001*			5.80	0.055
Localized	4.84	8.68		** **	1.10	1.71		
Regional	15.47	20.18			1.24	2.20		
Distant	52.59	65.29			4.04	5.48		
**TNLE classification**			93.38	< 0.001*			8.02	0.018*
≤4	13.49	18.15		** **	1.09	1.94		** **
<4, ≥8	20.25	28.53			2.12	3.47		
>8	44.57	53.67			4.41	5.13		
**LNR**	** **		132.69	< 0.001*			6.55	0.011*
≤0.04	14.44	19.48		** **	1.20	2.19		** **
>0.04	45.68	57.52			4.95	5.59		
**LODDS**			7.84	0.020*			3.18	0.204
≤-1.160	27.91	36.18		** **	2.69	3.52		** **
<-1.160, ≥-0.640	17.89	22.69			1.38	3.58		
*>-0.640*	17.76	23.82			1.71	2.22		
**PLNs (median)**	14.14	19.12	344.41	< 0.001*	1.22	2.22	41.72	0.010*
**LODDS (median)**	15.44	19.12	806.74	< 0.001*	0.00	0.00	733.98	< 0.001*
**LNR (median)**	14.14	19.12	535.12	< 0.001*	1.22	2.22	594.79	< 0.001*
**TNLE (median)**	23.22	33.43	111.73	0.934	5.22	5.22	38.58	0.996

Other, American Indian/AK Native, Asian/Pacific Islander; SEER, surveillance, epidemiology, and end results; LODDS: log ((the number of PLNs +0.5)/(the number of negative nodes +0.5)); LNR: the lymph node ratio; TNLE: total number of lymph nodes examined; PLNs: the number of positive lymph nodes; CSD, Cause-specific death; DOC, Death due to other causes; P value: log-rank test. * means statistically significant.

Ethnic origin and tumor size are prognostic risk factors for OS, in line with the grade related to CSS through competing univariate analysis. Demographics and clinicopathological prognosis factors *with p* < 0.05 were subsequently included in multivariate regression analysis. Through multivariate analysis, we found age at diagnosis, T stage, M stage, and SEER stage still significantly correlated with OS, while the ethnic origin and tumor size were not (**Table 3**).

**Table 3:** Multivariate analysis of continuous LODDS for OS and continuous LNR for CSS.

Variables	LODDS of OS			LNR of CSS		
	HR	95 %CI	P value	HR	95 %CI	P value
**Age at diagnosis**			< 0.001*			
<57	Reference			Reference		
57-67	1.048	0.833-1.318	0.587	1.010	0.791-1.29	0.940
>67	1.844	1.403-2.422	< 0.001*	1.250	0.880-1.790	0.210
**T stage**			< 0.001*			
1	Reference			Reference		
2	1.852	1.343-2.554	< 0.001*	1.840	1.296-2.63	0.001*
3	4.387	2.262-8.511	< 0.001*	4.420	2.064-9.49	< 0.001*
**M stage**			< 0.001*			
0	Reference			Reference		
1	2.057	1.734-3.625	< 0.001*	2.490	1.610-3.860	< 0.001*
**SEER stage**			0.018*			
Localized	Reference			Reference		
Regional	1.602	1.151-2.231	0.005*	1.810	1.253-2.620	0.002 *
Distant	1.713	0.827-3.552	0.148	1.960	0.849-4.540	0.110
**LODDS**	1.286	1.112-1.489	0.001*	NA	NA	NA
**LNR**	NA	NA	NA	1.890	1.15-3.11	0.012*
**Race**			0.103			
White	Reference			NA		
Black	1.589	1.024-2.466	0.039*	NA	NA	NA
Other	0.964	0.744-1.250	0.784	NA	NA	NA
**Grade**			NA			
I	NA			Reference		
II	NA	NA	NA	0.980	0.394-2.430	0.960
III	NA	NA	NA	1.300	0.549-3.060	0.550
IV	NA	NA	NA	0.990	0.411-2.390	0.980
**Tumor size, cm**			0.773			
<8.5	Reference			NA		
≥8.5	1.036	0.816-1.314	0.773	NA	NA	NA

Other, American Indian/AK Native, Asian/Pacific Islander; SEER, surveillance, epidemiology, and end results; LODDS: log ((the number of PLNs +0.5)/(the number of negative nodes +0.5)); LNR: the lymph node ratio; HR, hazard ratio; 95%CI, 95% confidence interval; OS, overall survival; CSS, cancer-specific survival; NA, not available; P value: log-rank test. * means statistically significant.

Multivariate competing-risk analysis indicated that only T stage (relative to T1 for T2: HR=1.840, for T3: HR=4.420), M stage (relative to M0 for M1: HR=2.490), SEER stage (relative to localized for regional: HR=1.810, for distant: HR=1.960) and LNR (HR=1.890) were significant prognostic factors affecting CSS.

### Comparison of four LN staging scheme models

Comparisons were made in order to select the most reliable lymph node factor from the four LN staging schemes. Based on the results of univariable analysis, PLNs, TNLE, LNR, and LODDS were separately intercalated into different multivariate regression models combined with independent factors (**Supplementary Tables 1–4**). For OS prognosis, LODDS (*continuous p* = 0.001 and classification *p* = 0.010) and LNR (continuous *p* < 0.001 and classification *p* = 0.001) were significant prognostic factors. PLNs and TNLE could not be used to determine OCCC prognosis. Please see the supplementary materials section **Tables 1**, **3** for further details.

Across the four LN staging schemes, only LNR (*continuous p* = 0.012) was an independent factor associated with CSS prognosis (please see **Supplementary Tables 2, 4**). To visually explore LODDS superiority over the other systems, C-index and AIC were calculated to compare the prognostic accuracy of LN staging models for overall survival in OCCC patients. Continuous LODDS had the highest C-index (0.634; 95% CI 0.603–0.666) and lowest AIC (-2400.96) in OS compared with the other staging systems (see **Supplementary Table 5**). As for the CSS model, the 3-year C-index of continuous LNR was comparatively better than the N stage (0.630 versus 0.625).

### Correlations between LODDS and LNR with independent clinicopathologic factors

According to the results of multivariate regression analysis, LODDS and LNR were selected as superior lymph node involvement variables for OS and CSS. **Figures 2A, B** demonstrate that as LODDS and LNR values increased, mortality rates also increased. The profile for survival time negatively correlated with LODDS (R=-0.19, *p <*0.001) and LNR (R=-0.22, *p <*0.001). Age at diagnosis, T stage, M stage, SEER stage, and continuous LODDS were identified for OS as independent factors. In the CSS model, only the T stage, M stage, SEER stage, and continuous LNR were significant predictors.

T stages were categorized into three subgroups in order to distinguish LODDS and LNR differences among the three groups. Boxplots showed that patients with larger tumor masses and extended tumor involvement tended to have higher LODDS and LNR values (**Figures 2D, G**). The greater scope of metastasized tumors, the higher LODDS and LNR would be (**Figures 2E, H**). LODDS and LNR values were consistently higher in patients with distant metastases than in those without distant metastases (**Figures 2F, I**). However, age at diagnosis only in OS highlighted differences between subgroups. Older patients, especially those over 67 years of age at diagnosis, had higher LODDS values compared to younger patients (*p* = 0.022, **Figure 2C**). LODDS for patients aged between 57-67 had no difference either in the younger or older groups (*p* > 0.05).

### Construction and validation of prognostic nomogram for OS and CSS

**Figure 3:**
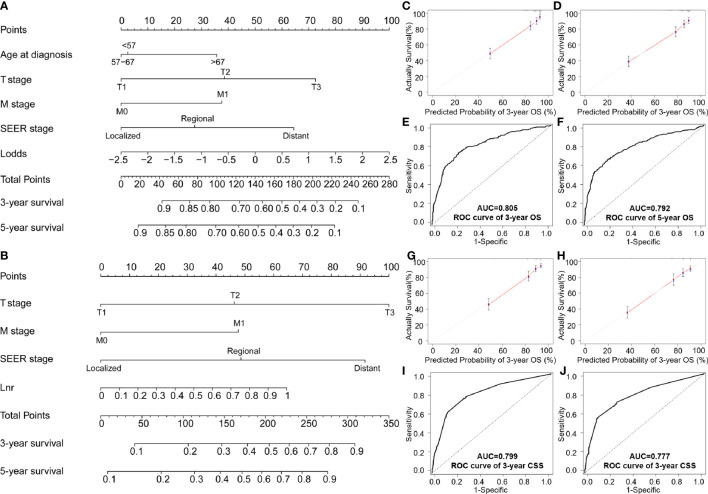
Nomogram testing of: **(A)** Overall survival; **(B)** CSS nomogram at predicting 3- and 5-year survival; **(C, D)** 3- and 5-year survival curves for OS; **(E, F)** ROC analysis of age, T stage, M stage, SEER stage and LODDS for OS; **(G, H)** 3- and 5-year calibration curves for predicting CSS; **(I, J)** ROC analysis of stage, M stage, SEER stage and LNR for CSS.

Nomograms for CSS and OS were constructed by incorporating prognostic variables from the OCCC training cohort. In total, age at diagnosis, T stage, M stage, SEER stage, and LODDS were included in the OS nomogram. T stage, M stage, SEER stage, and LNR were introduced into the CSS nomogram (**Figures 3A, B**). The nomogram improved prognostic performance compared to the 8th AJCC TNM staging system, according to C-index values. The respective C-index values for the OS and CSS nomograms in the training cohort were 0.759 and 0.791/0.784(3-year/5-year), respectively. These were superior to the traditional AJCC stage models at OCCC prognosis (OS: 0.733; CSS: 0.776/0.732[3-year/5-year]), as shown in **Supplementary Table 6**.

In the validation cohort, the C-index for the LODDS-based nomogram model of OS was 0.772. The LNR-based nomograms for CSS at 3-years (0.790) and 5-year (0.784) were also higher than the AJCC staging system. Similar results were observed in the ROC curves and with AUC values. High AUC values confirmed favorable sensitivity and specificity of the nomogram in both OS (3-year: 0.805; 5-year: 0.792) and CSS (3-year: 0.799; 5-year: 0.777), respectively (**Figures 3E, F, I, J**).

**Figure 4:**
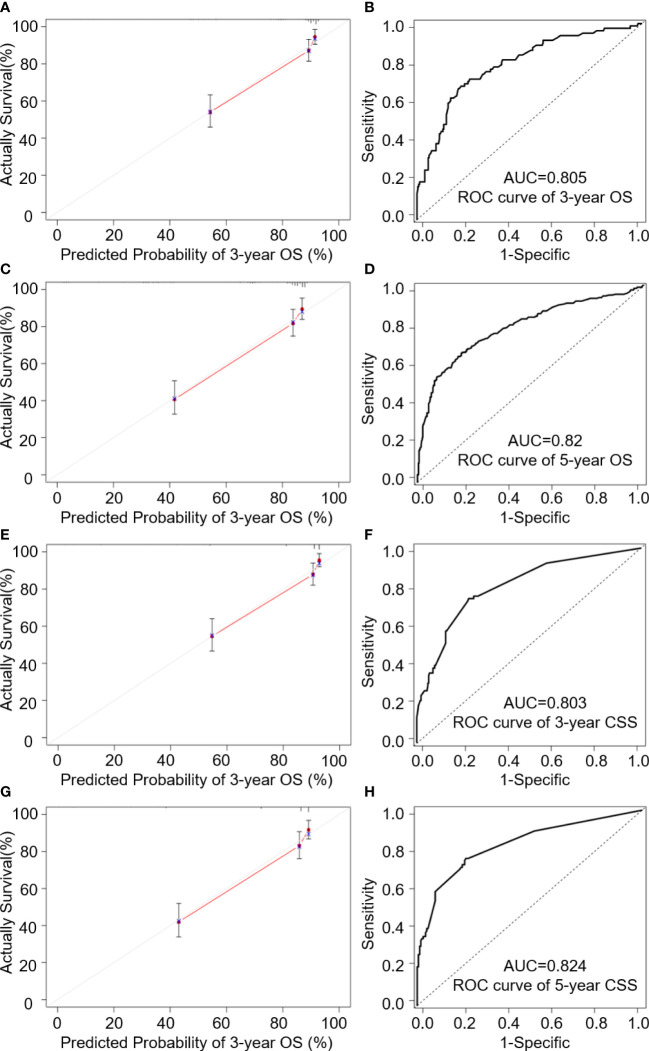
Internal validation cohort analysis of; **(A, C)** 3- and 5-year calibration curves of OS predictions; **(B, D)** 3- and 5-year ROC analysis of age, T stage, M stage and LODDS for OS; **(E, G)** 3- and 5-year calibration curves of CSS predictions; **(F, H)** ROC analysis of age, T stage, M stage, Seer stage and LNR for CSS.

AUC values in the validation cohort displayed improved prognostic accuracy compared to AUC values in the training cohort, especially the 3-year prognosis (OS: 0.82; CSS: 0.824). Please see **Figures 4B, D, F, H** for confirmation. In addition, calibration curves suggest good agreement between the optimal bootstrap predicted values and the actual survival rates of OCCC patients. This indicates appreciable prognostic reliability of the LODDS-based nomogram models (**Figures 3C, D, G, H**). Calibration curves also performed well for external validation cohorts in both the OS and CSS groups (**Figures 4A, C, E, G**).

## Discussion

Lymph node status is considered a key prognostic factor for ovarian clear cell carcinoma patients (20) although, there are a number of different parameters which can be assessed. Accurate staging of LN status can be used to generate survival predictions, which in turn enables us to develop postoperative treatment plans etc. (21) At present, the 8th UICC/AJCC N staging tool is the most frequently used; however, this may not account for complexities which could, if used correctly, increase prognostic specificity. The most recent UICC/AJCC N staging tool assesses lymph nodes status by identifying N0 and N1 patients, according to the presence (or absence) of regional lymph nodal metastases (22, 23). Unfortunately, this is also likely to be an oversimplification because this tool does not intercalate the TNLE during operations. In several comparisons of different lymph node staging systems, researchers have found that TNLE and PLNs are slightly less accurate than LNR and LODDS in different tumors (24–27). Although, few have comprehensively compared nodal staging systems to develop a necessary nomogram for ovarian clear cell carcinoma patients.

In this study, we analyzed anonymized data from 1,300 OCCC patients who underwent lymphadenectomy. Data were extracted from the SEER registry and retrospectively analyzed. Kaplan–Meier’s and log-rank curves were then used to assess the prognostic accuracy of different LN staging schemes. All the classified LN schemes including LODDS, LNR, TNLE, and the N stage had significant survival differences. In terms of continuous LN status variables, LNR was significant for overall survival prognosis while TNLE, PLNs, and LODDS were not. This is in line with previous research which has compared several lymph node assessment tools. The prognostic benefit of different LN models has also been reported to increase the quality of LN assessment in various other cancers (28, 29). However, we did not rank these because the objective of this study was to develop a nomogram which can discern patients and provide more individualized prognoses. Other researchers may find it necessary to rank these factors, as we move toward artificial intelligence-based prognostics. It would seem imprudent to remove potentially important factors without first ranking these using a larger sample.

Analysis using the C-index suggests that continuous LODDS and continuous LNR were superior, having the strongest predictive accuracy for both overall survival and cancer-specific survival. Previous studies have shown that prognostic nomograms can be used to facilitate prognostic assessments and can be used to develop more personalized care (30, 31). In this study, calibration curves for 3- and 5-year survival and CSS matched well with the ideal line. We also generated time-dependent ROC curves to estimate sensitivities and specificities at 3- and 5-year survival points for both OS and CSS. Findings suggest that our novel nomogram is more accurate compared to both the AJCC tool and SEER staging, according to C-index analysis. This means, our nomogram has statistical significance and clinical precision compared with other more traditional staging systems. Although in the future, we must use more real-world data to further verify the accuracy and predictive ability of these models.

This study suggests that LNR and LODDS are superior prognostic predictors for OCCC patients under the nomogram model. This finding is supported by other studies which submit that LNR and LODDS could be useful in guiding prognostics for gastric, rectal, pancreatic, breast, and esophageal cancer, as well as a number of other cancers (29, 32–35). Although, evidence is conflicting around which is superior. For example, Wang et al. found that the LODDS system was significantly better than TNLE for colorectal cancer (36). While other research into pancreatic ductal adenocarcinoma, has found that while LNR might provide more accurate prognosis although, it is limited and increases in strength with TNLE (37). LODDS has also been found to be better for several tumors. In penile cancer (38), LODDS (log-likelihood = 3832 vs. 3798; p < 0.001) had better prognostic performance than pN and better discriminability than lymph node density (AIC = 3902 vs. 3928).

In addition, Gu et al. (39) showed that the LODDS staging system was superior to other lymph-node classifiers for the prognosis of patients undergoing gastrectomy for gastric cancer (GC), which could be incorporated into a GC staging system. However, in stage III colorectal cancer, LNR seemed more promising to predict the postoperative outcomes (40). Therefore, questions remain around LODDS and LNR and whether there might be a specific N stage marker for specific neoplasia. Notably, nomograms based on the LN scheme seem to provide a vital predictive element for a number of cancers, which can effectively guide patient prognoses.

Based on LODDS, LNR, and other prognosis-related parameters, we established a novel prognostic model where patients were randomly assigned to either training or validation cohorts to avoid selection bias, to predict OS and CSS for OCCC. It is worth mentioning that this was the first study to compare and contrast nodal staging tools for OCCC patients. The nomogram showed relatively high accuracy, with the C-index exceeding 0.7 in both the training cohort and the validation cohort. Compared to the widely utilized FIGO staging system, LODDS-based and LNR-based nomograms in OCCC patients possess superior predictive ability. However, a high AUC does not equate to high clinical applicability of this prediction model. Therefore, we introduced calibration curves to assess the clinical validity of our nomogram using OS and CSS for OCCC patients after surgery. We found for CSS, it was necessary to take a multi-state competitive risk model into consideration to guide survival analysis, including competing outcome events. We also found, unlike previous studies, that there was a need to classify different lymph node staging schemes into either a continuous classification or categorical classification. Once we adjusted our approach, we were able to compare the more subtle differences in lymph node status. We also found that LNR and LODDS based nomograms were able to more accurately predict OCCC outcomes.

From a methodological perspective, the SEER registry is an excellent resource, providing researchers with a huge sample of data across various types of cancers. The National Cancer Institute has provided the world with a wonderful opportunity for training and gaining insight into changing cancer prevalence, mortality with several linked data sources. This open-access source is a model for other countries wishing to enable researchers from various fields to retrospectively investigate shifting trends and correlations. However, there are a number of issues which ought to be mentioned. Firstly, the SEER registry does not provide complete patient data and therefore any analysis will be a little superficial. For example, little detailed information around chemotherapeutics and radiotherapies is provided. This means, we are unable to assess adherence to a chosen regimen and unable to intercalate dose-intensity or patterns in the timing of adjuvant interventions. We feel this would certainly improve any future open-access cancer registry. We also feel that countries such as China, should develop a similar program so that we can make more sophisticated comparisons which will help us to consider the minutia involved in any prescribed regimen for all cancers.

Before providing recommendations, it is necessary to consider the limitations of this study. We have already discussed, the deficiencies in the SEER database which we may overcome in the future. However, one of the major difficulties in this type of research is that it is retrospective. This means, there are a number of shortcomings but that does not negate the usefulness of this research. We have to remember this type of research is exploratory and findings will only ever be used to generate further prospective studies. Of course, we need further clinical studies to investigate the value of these nomograms. Specifically, larger sample, prospective, randomized controlled trials would be useful as a next step. This study did however clarify the current status of ovarian clear cell carcinoma and improve prognostic predictability. This study also found that age at diagnosis, T stage, M stage, and SEER stage were all independent prognostic factors for OS and CSS in ovarian clear cell carcinoma patients. This provides a springboard for further research and nomogram development, which is essential for cancer care.

## Conclusions

LODDS and LNR are more prognostically useful than AJCC/UICC N and other lymph node staging systems for ovarian clear cell carcinoma patients undergoing lymphadenectomy. Our LODDS-based nomogram and the LNR-based nomogram ensured superior stratification of patients with nodal metastases for both OS and CSS. We can also tentatively recommend these nomograms for future clinical practice. Although, further prospective research is required across a large Chinese population. Finally, we recommend a SEER type registry should be developed in China for the Chinese population which is nuanced and distinct from the US population. We would hope that any new open-access registry will include more sophisticated details around regimens and adherence so that we can gain the insight into the effects of adjuvant treatments and timing.

## Data availability statement

The datasets presented in this study can be found in online repositories. The names of the repository/repositories and accession number(s) can be found below: https://seer.cancer.gov/data/.

## Ethics statement

Ethical review and approval was not required for the study on human participants in accordance with the local legislation and institutional requirements. Written informed consent for participation was not required for this study in accordance with the national legislation and the institutional requirements.

## Author contributions

YLL, and LX were responsible for the design, data analysis, and critical revision of the manuscript. MYM, and XYL completed the data collection. SS, KXL, and YW helped to draft and critically revise this report. WC, SL, and YXW contributed to helpful discussion and reviewed the manuscript.

## Funding

The work was supported by the National Natural Science Foundation of China (No. 82072361) and Beijing Chaoyang District Science and technology Plan (CYSF2030).

## Acknowledgments

The authors acknowledge the efforts of the Surveillance, Epidemiology, and End Results (SEER) Program tumor registries in the creation of the SEER database. The interpretation and reporting of these data are the sole responsibility of the authors.

## Conflict of interest

The authors declare that the research was conducted in the absence of any commercial or financial relationships that could be construed as a potential conflict of interest.

## Publisher’s note

All claims expressed in this article are solely those of the authors and do not necessarily represent those of their affiliated organizations, or those of the publisher, the editors and the reviewers. Any product that may be evaluated in this article, or claim that may be made by its manufacturer, is not guaranteed or endorsed by the publisher.
