# Supplementary material for: Developing and validating a prognostic nomogram for ovarian clear cell carcinoma patients: A retrospective comparison of lymph node staging schemes with competing risk analysis
Source: Front Oncol. 2022 Nov 9;12:940601. doi: 10.3389/fonc.2022.940601 (PMC9682100; doi:10.3389/fonc.2022.940601)

Supplementary Material

## Supplementary Figures

**Figure S1.** Flowchart of ovarian clear cell carcinoma patient search and selection from the SEER database


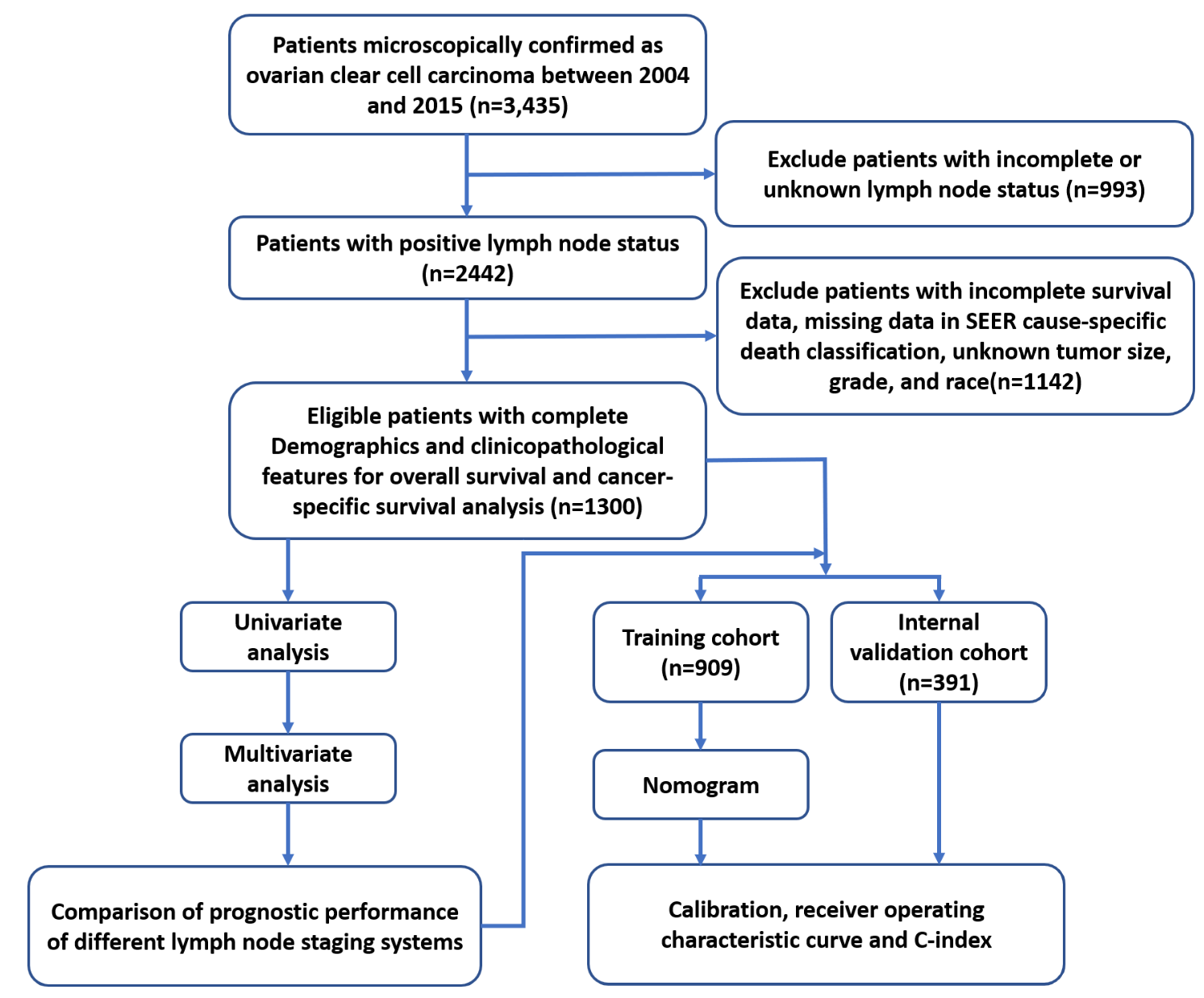


**Figure S2.** Kaplan–Meier curves for overall survival and cumulative incidence function curves for cancer-specific deaths stratified by prognostic factors based on (A-B) Age at diagnosis; (C-D); Year at diagnosis; (E-F) Ethnic origin; (G-H) Grade; (I-J) Size; (K-L) T stage; (M-N) M stage; and (O-P) SEER stage


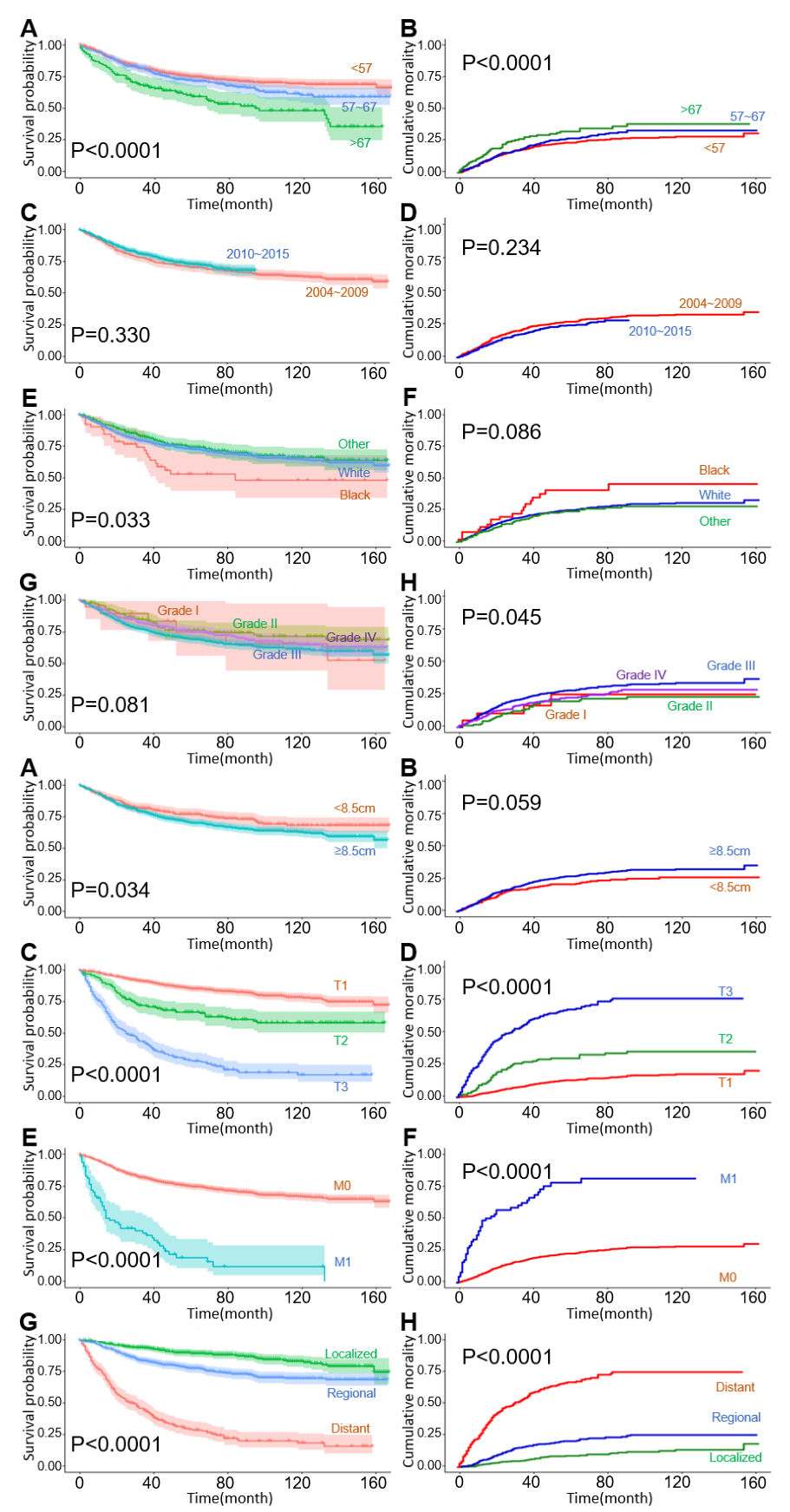

Supplement: Supplementary file 1 [file DataSheet_1.docx]
